# Minimal effect of sleep on the risk of age-related macular degeneration: a Mendelian randomization study

**DOI:** 10.3389/fnagi.2023.1159711

**Published:** 2023-08-21

**Authors:** Rong-Cheng Zhu, Fen-Fen Li, Yi-Qing Wu, Quan-Yong Yi, Xiu-Feng Huang

**Affiliations:** ^1^Zhejiang Provincial Clinical Research Center for Pediatric Disease, The Second Affiliated Hospital and Yuying Children’s Hospital of Wenzhou Medical University, Wenzhou, Zhejiang, China; ^2^National Clinical Research Center for Ocular Diseases, Eye Hospital, Wenzhou Medical University, Wenzhou, China; ^3^State Key Laboratory of Ophthalmology, Optometry and Visual Science, Eye Hospital, Wenzhou Medical University, Wenzhou, China; ^4^Department of Pediatrics, The Second School of Medicine, The Second Affiliated Hospital and Yuying Children’s Hospital of Wenzhou Medical University, Wenzhou, Zhejiang, China; ^5^The Affiliated Ningbo Eye Hospital of Wenzhou Medical University, Ningbo, Zhejiang, China

**Keywords:** Mendelian randomization, causal inference, age-related macular degeneration, sleep duration, genetic instrument

## Abstract

**Aims:**

Observational studies have shown that sleep pattern is associated with age-related macular degeneration (AMD), but whether sleep pattern is a causal factor for AMD remains unclear. This study aims to use Mendelian randomization (MR) analysis to investigate the potential causal relationship between sleep traits and AMD.

**Methods:**

This is a two-sample MR study. The single-nucleotide polymorphisms associated with AMD and early AMD were selected as the outcome from two different genome-wide association studies (GWAS): the early AMD GWAS with 14,034 cases and 91,214 controls, and AMD GWAS with 3,553 cases and 147,089 controls. The datasets of sleep duration, daytime dozing, and sleeplessness were used as exposure, which comprised nearly 0.46 million participants. Inverse-variance weighted method was used as the main result, and comprehensive sensitivity analyses were conducted to estimate the robustness of identified associations and the impact of potential horizontal pleiotropy.

**Results:**

Through MR analysis, we found that sleep duration was significantly associated with AMD (OR = 0.983, 95% CI = 0.970–0.996, *P*-value = 0.01). We also found suggestive evidence for the association of genetically predicted sleep duration with early AMD, which showed a consistent direction of effect with a marginal significance (OR = 0.724, 95% CI = 0.503–1.041, *P*-value = 0.08). Sensitivity analyses further supported the robustness of the causal relationship between sleep duration and AMD. However, we were unable to determine the relationship between daytime dozing or sleeplessness and AMD (including early AMD) (*P*-value > 0.05).

**Conclusion:**

Sleep duration affects the causal risk for AMD; that is, longer sleep duration reduces the risk of AMD, while shorter sleep duration increases the risk of AMD. Although the influence is minimal, keeping adequate sleep duration is recommended, especially for patients with intermediate or advanced AMD.

## 1. Introduction

Age-related macular degeneration (AMD) is the leading cause of irreversible visual impairment in the aging population, estimated to be around 300 million people worldwide by 2040 (Wong et al., 2014). Visual impairment caused by AMD decreases the quality of life and burdens society heavily (Moshfeghi et al., 2020). The progression of AMD is classified as early, intermediate, and late stage, according to the severity of fundus lesions, such as drusen size and pigmentary abnormalities (Ferris et al., 2013). Although significant advances have been made in understanding AMD, the pathogenesis of AMD remains partly unclear (Mitchell et al., 2018). Therefore, recognizing causal risk factors for AMD is critical for its prevention and treatment.

It is widely known that genetic and environmental factors are involved in the development of AMD. Enormous efforts have been made to explore the impact of environmental and lifestyle factors on AMD; thus, multiple risk factors have been reported, such as smoking, obesity and dietary (Sobrin and Seddon, 2014). However, the relationship between sleep pattern and AMD is still an unanswerable question. There is no unanimous conclusion as to whether sleep pattern influences the risk of AMD. A recent study investigating the incidence of AMD in 8,225 patients observed that patients with insomnia were 33% more likely to have subsequent AMD (HR = 1.33; 95% CI = 1.18–1.48) (Tsai et al., 2020). On the contrary, another study failed to detect an association with long sleep (more than 8 h) in 316 patients with nAMD compared to 500 patients without AMD (Khurana et al., 2016).

Mendelian randomization (MR) approach has proven to be a powerful methodology for investigating the putative causal relationships between risk factors and AMD, as its principles and purpose are similar to randomized control trial (RCT) (Davies et al., 2018). Compared to traditional observational studies, MR analysis is less likely to be disturbed by confounding factors or reverse causation (Davies et al., 2018; Pingault et al., 2018). Previously, two MR studies have reported a causal relationship between increased high-density lipoprotein cholesterol (HDL-C) levels and advanced AMD risk (Burgess and Davey Smith, 2017; Fan et al., 2017). Another study evaluated smoking, alcohol consumption, blood pressure, body mass index, and glycemic risk factors with AMD and found a potential causal association of alcohol consumption with an increased risk of geographic atrophy, smoking initiation, and lifetime smoking with an increased risk of advanced AMD, and smoking cessation with a decreased risk of advanced AMD (Kuan et al., 2021). Therefore, we adopted a two-sample MR approach to determine the causal relationship between sleep traits and AMD.

## 2. Materials and methods

### 2.1. Study design

**FIGURE 1:**
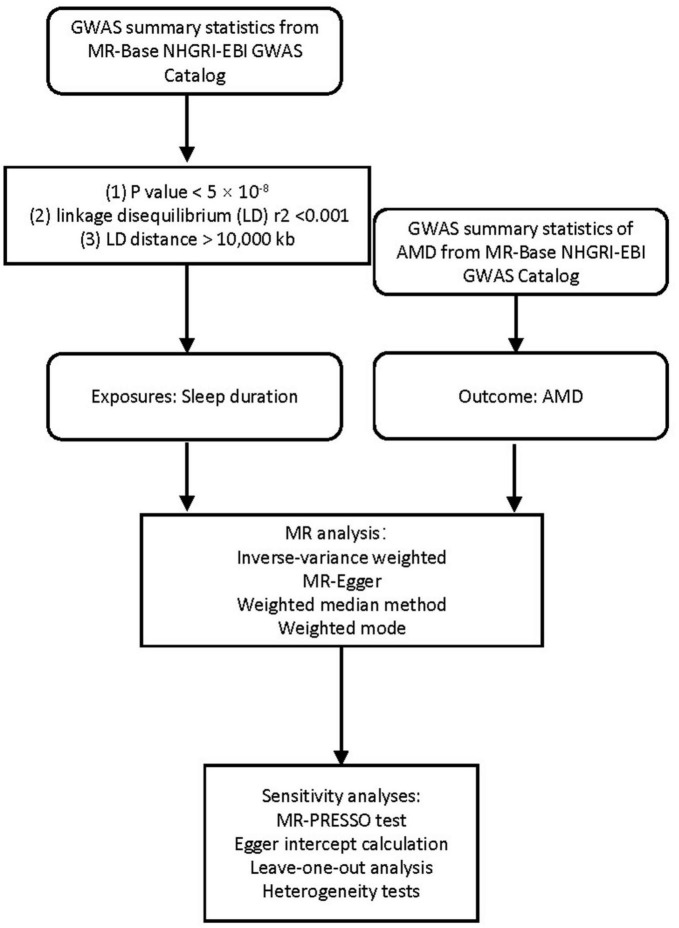
Flowchart of this MR study.

We employed a two-sample MR design using summary estimates to examine the lifelong effect of sleep duration on genetic liability to AMD (Figure 1), allowing GWAS summary statistics for exposure and outcome from independent studies. Three assumptions are required to obtain an unbiased causal effect estimate (Supplementary Figure 1; Lawlor et al., 2008). First, the genetic instrument is strongly associated with the exposures. Second, the genetic instrument does not influence the outcome through some pathway other than exposure. Third, the genetic instrument does not associate with confounders of the exposure-outcome relationship.

### 2.2. GWAS summary statistics for sleep duration and AMD

Genome-wide association studies summary statistics of sleep duration and AMD were obtained from MR-Base NHGRI-EBI GWAS Catalog^1^ (Dashti et al., 2019). Concerning the clinical diversity, summary data from two different GWAS were selected as the outcome: the early AMD GWAS (ebi-a-GCST010723) with 14,034 cases and 91,214 controls (11,304,110 SNPs), and AMD GWAS (ukb-b-17194) with 3,553 cases and 147,089 controls (9,851,867 SNPs). The datasets of sleep duration (ukb-b-4424), daytime dozing (ukb-b-5776), and sleeplessness (ukb-b-3957) were used as exposure, which comprised 460,099, 460,913, and 462,341 participants, respectively (9,851,867 SNPs). All five datasets mentioned were derived from the European population. The details have been summarized in Table 1. This study only used publicly available data, and the relevant ethical approval can be found in the corresponding studies.

**TABLE 1:** Description of the GWAS summary statistics of traits.

Trait	GWAS Catalog accession number	Sample size	Number of SNPs	Consortium	Author	Year	Population
Sleep duration	ukb-b-4424	460,099	9,851,867	MRC-IEU	Ben Elsworth	2018	European
Daytime dozing	ukb-b-5776	460,913	9,851,867	MRC-IEU	Ben Elsworth	2018	European
Sleeplessness	ukb-b-3957	462,341	9,851,867	MRC-IEU	Ben Elsworth	2018	European
Age-related macular degeneration	ukb-b-17194	150,642	9,851,867	MRC-IEU	Ben Elsworth	2018	European
Early age-related macular degeneration	ebi-a-GCST010723	105,248	11,304,110	NA	Winkler TW	2020	European

N/A, not available.

### 2.3. MR analysis

Mendelian randomization analysis between exposures (sleep duration, daytime dozing, and sleeplessness) and outcomes (AMD and early AMD) was performed using the TwoSampleMR v0.5.5 package (Hemani et al., 2018). We applied the following standards to the selection of independent genome-wide significant variants as a genetic tool for sleep pattern: (1) *P*-value on sleep duration <5 × 10^–8^; (2) linkage disequilibrium (LD) *r*^2^ < 0.001; (3) LD distance >10,000 kb. Inverse-variance weighted (IVW) method is primarily intended to estimate the effect of instrumental variables on outcomes (Burgess et al., 2013; Hemani et al., 2018), and it is secure to utilize in a single large-scale data set (Minelli et al., 2021). Therefore, in this study, the IVW method was chosen as the main method of estimating the association between sleep traits and AMD (including early AMD).

### 2.4. Sensitivity analysis

To further explore the robustness of the association between sleep traits (including sleep duration, daytime dozing, and sleeplessness) and AMD (including early AMD), we chose the following approach to perform a holistic sensitivity analysis. We used three additional approaches based on the TwoSampleMR R package for sensitivity analysis, including MR-Egger regression (Bowden et al., 2015), Weighted median method (Bowden et al., 2016), and Weighted mode (Hemani et al., 2018), which tolerate the presence of horizontal pleiotropy but have less statistical power than IVW. In addition, we also estimated the robustness of identified associations and the impact of potential horizontal pleiotropy using MR pleiotropy residual sum and outlier (MR-PRESSO) test (Verbanck et al., 2018), Egger intercept calculation (Bowden et al., 2015), Leave-one-out analysis (Hemani et al., 2018), and Heterogeneity tests (Corlin et al., 2021).

## 3. Results

### 3.1. Causal effect of sleep duration on AMD

**FIGURE 2:**
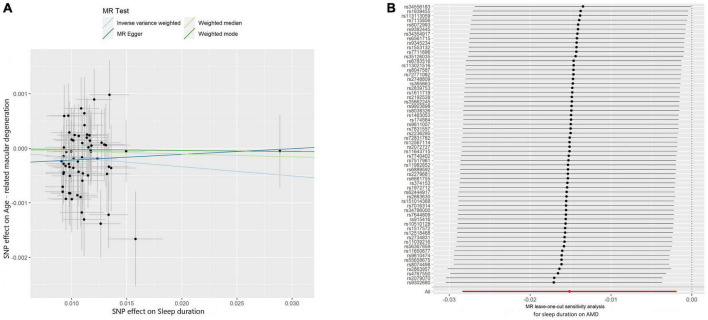
Scatter and leave-one-out plots of sleep duration with the risk of AMD. **(A)** Scatter plot demonstrating the effect of sleep-associated genetic variants on AMD on the log-odds scale. The slopes of each line represent the causal association for each method. Scatter plots were utilized to display per-allele association with outcome risk in relation to per-allele association with one standard deviation of exposure. Vertical and horizontal gray lines were included to show the 95% CI for each SNP. **(B)** Leave-one-out analysis for IVW MR of sleep duration on AMD in summary-level analyses and to assess the effect of each SNP in driving causality.

The IVW method was used to assess the causal effect of sleep traits on AMD. The MR estimates showed that sleep duration played a protective role in AMD (OR = 0.983, 95% CI = 0.970–0.996, *P*-value = 0.01; Table 2). The Scatter plot of the effect of sleep duration on AMD was shown in Figure 2A. Both the Weighted median and Weighted mode methods supported that the sleep duration had a protective effect on AMD (OR < 1), although not statistically significant (Table 2). MR Egger method showed an opposite direction without statistical significance. These results indicate that sleep duration is a protective factor for AMD (Figure 2B).

**TABLE 2:** Casual effect of sleep duration on AMD and early AMD.

Exposure	SNPs	Method	OR	95% CI	*P*-value
Age-related macular	63	**Inverse variance weighted**	**0.983**	**0.970–0.996**	**0.01**
		MR Egger	1.010	0.952–1.073	0.74
		Weighted median	0.995	0.976–1.015	0.61
		Weighted mode	0.998	0.965–1.032	0.91
Early age-related macular degeneration	69	Inverse variance weighted	0.724	0.503–1.041	0.08
		MR Egger	0.898	0.220–3.660	0.88
		Weighted median	0.625	0.379–1.030	0.06
		Weighted mode	0.559	0.228–1.367	0.21

OR, odds ratio; 95% CI, 95% confidence interval. Significant associations (*P*-value < 0.05) are highlighted in bold format.

Comprehensive sensitivity analyses were performed to assess the robustness of the causal relationship between sleep duration and AMD. In the Egger intercept calculation test, no evidence of directional horizontal pleiotropy effects was found as the Egger intercept was very close to zero and the *P*-value > 0.05 (intercept = −0.0003, *P*-value = 0.365). The MR-PRESSO test also suggested that no horizontal pleiotropic outliers distort our result (*P*-value of Global Test = 0.89; Table 3). Moreover, no outliers affected the relationship based on the leave-one-out analysis (Figure 2). In addition, the heterogeneity test further confirmed the absence of significant horizontal pleiotropy and heterogeneities (*P*-value of IVW method: 0.878; *P*-value of MR Egger method: 0.878). Taken together, results from sensitivity analysis further support that sleep duration has a protective effect on the risk of AMD.

**TABLE 3:** MR-PRESSO estimates of the associations between sleep duration and AMD and early AMD.

Exposure	MR analysis	Causal estimate	*P*-value	Global test *P*-value	Distortion *P*-value
Age-related macular	Raw	−0.017	0.006	0.890	NA
	Outlier-corrected	NA	NA		
Early age-related macular degeneration	Raw	−0.323	0.086	0.100	NA
	Outlier-corrected	NA	NA		

N/A, not available.

### 3.2. Causal effect of sleep duration on early AMD

**FIGURE 3:**
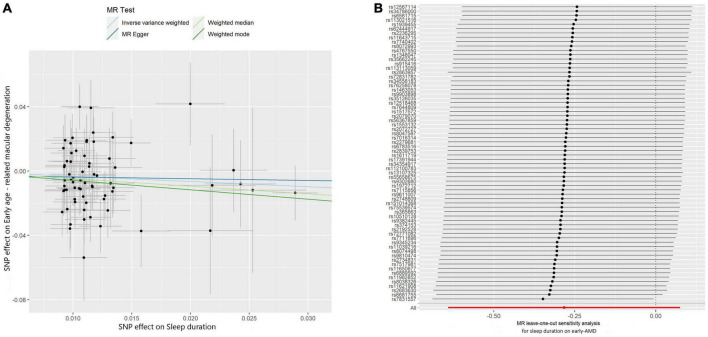
Scatter and leave-one-out plots of sleep duration with the risk of early AMD. **(A)** Scatter plot demonstrating the effect of sleep-associated genetic variants on early AMD on the log-odds scale. The slopes of each line represent the causal association for each method. Scatter plots were utilized to display per-allele association with outcome risk in relation to per-allele association with one standard deviation of exposure. Vertical and horizontal gray lines were included to show the 95% CI for each SNP. **(B)** Leave-one-out analysis for IVW MR of sleep duration on early AMD in summary-level analyses and to assess the effect of each SNP in driving causality.

Subsequently, we investigated the effect of sleep duration on early AMD as a sub-analysis. Interestingly, all four MR methods consistently showed that longer sleep duration reduces the risk of AMD (OR < 1, Table 2; Figure 3A). However, the protective effect of sleep duration on early AMD was observed with only a marginal significance (IVW method: *P*-value = 0.08; Weighted median method, *P*-value = 0.06; Table 2). Sensitivity analysis was also conducted. Egger intercept calculation test showed no evidence of directional horizontal pleiotropy effects (intercept = −0.0026, *P*-value = 0.756). MR-PRESSO test suggested no horizontal pleiotropic outliers (Table 3). Leave-one-out analysis showed no outliers among 69 SNPs (Figure 3B). Heterogeneity test suggested no significant horizontal pleiotropy and heterogeneities (*P*-value of IVW method: 0.087; *P*-value of MR Egger method: 0.075). This sub-analysis showed a consistent direction of effect with the discovery study, indicating that sleep duration is a protective factor for AMD.

### 3.3. Study of daytime dozing or sleeplessness

For daytime dozing, we found the same direction of effect size in IVW, Weighted median, and Weighted mode while the opposite direction in MR Egger (OR < 1), and none of them were significant (*P* > 0.05). Although the effect directions of daytime dozing for early AMD were similar in all four methods (OR < 1), none of their results were significant (Supplementary Table 1; Supplementary Figure 2). In the study on sleeplessness, no statistically significant outcomes were observed in either AMD or early AMD (*p* > 0.05) (Supplementary Table 2; Supplementary Figure 3).

## 4. Discussion

In this study, we explored that less sleep duration increased the risk of AMD (OR = 1.02, *P*-value = 0.01). We also found suggestive evidence for the association of genetically predicted sleep duration with early AMD, which showed a consistent direction of effect but with a marginal significance (*P*-value = 0.08). One possible explanation is that early AMD has a higher heritability than intermediate or advanced AMD, leading to less influence from environmental or lifestyle factors. Evidence from this study recommended that AMD patients should keep adequate sleep duration, especially those with intermediate or advanced AMD. However, for sleeplessness and dozing, we could not yet find a relationship between them and AMD (*p*-value > 0.05).

To date, evidence from observational studies showed a remarkable heterogeneity in the association of different sleep duration with AMD. Previously, an observational study on 57 patients with neovascular AMD (nAMD) compared to 108 controls found a significantly increased risk of nAMD in patients sleeping less than 6 h compared to those sleeping 7–8 h (OR = 3.29; 95% CI = 1.32–8.27) (Perez-Canales et al., 2016). On the contrary, another study failed to detect an association with long sleep in 316 patients with nAMD compared to 500 patients without AMD (Khurana et al., 2016). Besides, a MR study based on the neurodegenerative disorders, including age-related macular degeneration (AMD), Alzheimer’s disease (AD), amyotrophic lateral sclerosis (ALS), multiple sclerosis (MS), and Parkinson’s disease (PD), have not revealed relationships among the sleep duration and AMD (IVW *P*-value = 0.15) (Grover et al., 2022). In the current study, we found that sleep duration influences the risk of AMD, that is, longer sleep duration reduces the risk of AMD while shorter sleep duration increases the risk of AMD. Following reason can explain why our study found a significant association between sleep duration and AMD but the previous MR study failed. First, the sample size was five-fold increased in this study (150,642) than others (33,976) (Grover et al., 2022). Second, it’s worth noting that the sleep duration only have minimal effect on AMD (OR = 0.983, 95% CI = 0.970–0.996). In this study, AMD has an OR value of 0.983 and early AMD has an OR value of 0.724. The difference in OR between AMD and early AMD can be attributed to several plausible explanations. Firstly, early AMD signifies the initial stages of the disease, while AMD represents a more advanced and severe form. As the disease progresses, the risk factors may evolve, resulting in varying OR values. Secondly, disparities in sample sizes and patient characteristics between the two groups could significantly influence the observed differences in OR values. Thirdly, divergent treatment and management strategies for early AMD and AMD might also impact the development and progression of the disease. Nevertheless, evidence from this study may help solve the debate on the associations between sleep duration and AMD. Thus, our results provide valuable evidence to support the previous observational study that insufficient sleep time correlated with the onset of subsequent AMD (Tsai et al., 2020).

Daytime dozing is a manifestation of abnormal sleep cycles and dysregulation of circadian rhythms increases the activity of the WNT/β-catenin pathway, which is associated with the development of AMD (Vallee et al., 2020). In contrast to our hypothesis, our study did not find an association between daytime sleepiness and AMD, perhaps due to a protective role played by increased daytime melatonin secretion (Schmid-Kubista et al., 2009). Sleeplessness not only reduces sleep duration but also diminishes sleep quality and makes it difficult to fall asleep. As a result, it has more severe consequences and impairments beyond a mere reduction in sleep duration (Roth, 2007). Previous studies have identified sleeplessness as a risk factor for the development of age-related macular degeneration (AMD) (Tsai et al., 2020). However, our study did not find significant evidence that sleeplessness was associated with AMD. It is possible that our study had an insufficient sample size or that previous studies did not accurately distinguish between sleeplessness and reduced sleep duration, including a sample that had only reduced sleep. In our study, we used MR methods to eliminate the effects of confounding factors such as sleep quality and sleep cycles so that our final results are not influenced by other confounding factors and have some clinical significance, even though we have no significant evidence that dozing, insomnia and AMD are related.

As an important environmental and lifestyle factor, how sleep pattern plays a role in disease as well as in health has been increasingly under investigation. It has been observed that sleeping is not only associated with many systemic diseases, such as hypertension (Levenson et al., 2017) and coronary heart disease (Cheng et al., 2014; Lao et al., 2018), but also with eye diseases, such as diabetic retinopathy (Tan et al., 2018), vision impairment (Sun et al., 2021), dry eye (Au et al., 2019; Zheng et al., 2022), myopia and cataract (Zhou et al., 2023). Interestingly, researchers provided evidence that night sleeping hours were associated with the decreased expression of TIMP-3, IER3, and SLC16A8 in AMD patients (Sharma et al., 2021). In addition disrupted sleep patterns also promote AMD due to the production of reactive oxygen species, a lack of oxygen and inflammatory markers, such as C-reactive protein (CRP) and interleukin-6 (IL-6) (Colak et al., 2012; Irwin, 2019; Han et al., 2021). Theoretically, alteration of sleep duration might affect AMD by the darkness-stimulated melatonin synthesis (Tosini et al., 2012) and impairment of the vitreous pump and glymphatic system (Magonio, 2022). It has been postulated that melatonin may protect retinal cells from oxidative stress and improve mitochondrial function. Disruptions to this circadian clock could impair retinal homeostasis and contribute to AMD progression. In addition, during sleep, the glymphatic system is believed to facilitate the clearance of neurotoxic waste products from the brain, including the retina. Impaired glymphatic clearance due to sleep disturbances may lead to the accumulation of toxic metabolites, potentially contributing to retinal degeneration (Chong et al., 2022).

There are limitations in this study. First, data of exposure and outcome are from European populations, where the incidence of AMD is significantly higher than in other populations, so it is questionable whether the results can be applied to other populations. Second, our sleep duration is self-reported, which is less accurate and objective than the results obtained from polysomnography. Regardless, it is believed that self-reported sleep duration has an acceptable correlation with the results obtained from polysomnography (Cespedes et al., 2016). Third, the magnitude of the effect in this study is relatively small. The MR findings only reflect the change in AMD risk due to a genetically predisposed (lifetime) sleep duration. Thus, we can speculate that the effect will be larger using real RCT design.

In conclusion, our results suggest that sleep duration affects the causal risk for AMD; that is, longer sleep duration reduces the risk of AMD while shorter sleep duration increases the risk of AMD. Although the influence is minimal, keeping adequate sleep duration is recommended, especially for patients with intermediate or advanced AMD.

## Data availability statement

The original contributions presented in this study are included in this article/Supplementary material, further inquiries can be directed to the corresponding authors.

## Author contributions

X-FH, Q-YY, and F-FL contributed to the study design. X-FH, R-CZ, and Y-QW contributed to data collection and analysis. R-CZ and F-FL wrote the manuscript. X-FH and Q-YY revised the manuscript. All authors contributed to the article and approved the submitted version.

## Footnotes

1
https://gwas.mrcieu.ac.uk/
