# Supplementary material for: Minimal effect of sleep on the risk of age-related macular degeneration: a Mendelian randomization study
Source: Front Aging Neurosci. 2023 Aug 21;15:1159711. doi: 10.3389/fnagi.2023.1159711 (PMC10475584; doi:10.3389/fnagi.2023.1159711)
Supplement: Supplementary file 1 [file Data_Sheet_1.PDF]

Table S1. Casual effect of Daytime dozing on AMD and early AMD.

Exposure	SNPs	Method	OR	95% CI	*P*-value
**Age-related macular degeneration**	30	Inverse variance weighted	1.024	0.993-1.056	0.13
		MR Egger	0.997	0.834-1.191	0.97
		Weighted median	1.021	0.980-1.064	0.33
		Weighted mode	1.030	0.959-1.105	0.43
**Early age-related macular degeneration**	30	Inverse variance weighted	0.884	0.387-2.021	0.77
		MR Egger	0.36	0.008-17.092	0.21
		Weighted median	0.50	0.166-1.502	0.22
		Weighted mode	0.249	0.034-1.826	0.18

OR, odds ratio; 95% CI, 95% confidence interval.

Significant associations (*P* value < 0.05) are highlighted in bold format.

Table S2. Casual effect of Sleeplessness on AMD and early AMD.

Exposure	SNPs	Method	OR	95% CI	*P*-value
**Age-related macular degeneration**	35	Inverse variance weighted	1.016	0.997-1036	0.10
		MR Egger	1.027	0.905-1.166	0.68
		Weighted median	1.009	0.983-1.036	0.49
		Weighted mode	0.999	0.940-1.062	0.98
**Early age-related macular degeneration**	39	Inverse variance weighted	0.614	0.304-1.239	0.17
		MR Egger	0.257	0.024-2.713	0.27
		Weighted median	0.533	0.259-1.094	0.09
		Weighted mode	0.548	0.151-1.987	0.37

OR, odds ratio; 95% CI, 95% confidence interval.

Significant associations (*P* value < 0.05) are highlighted in bold format.

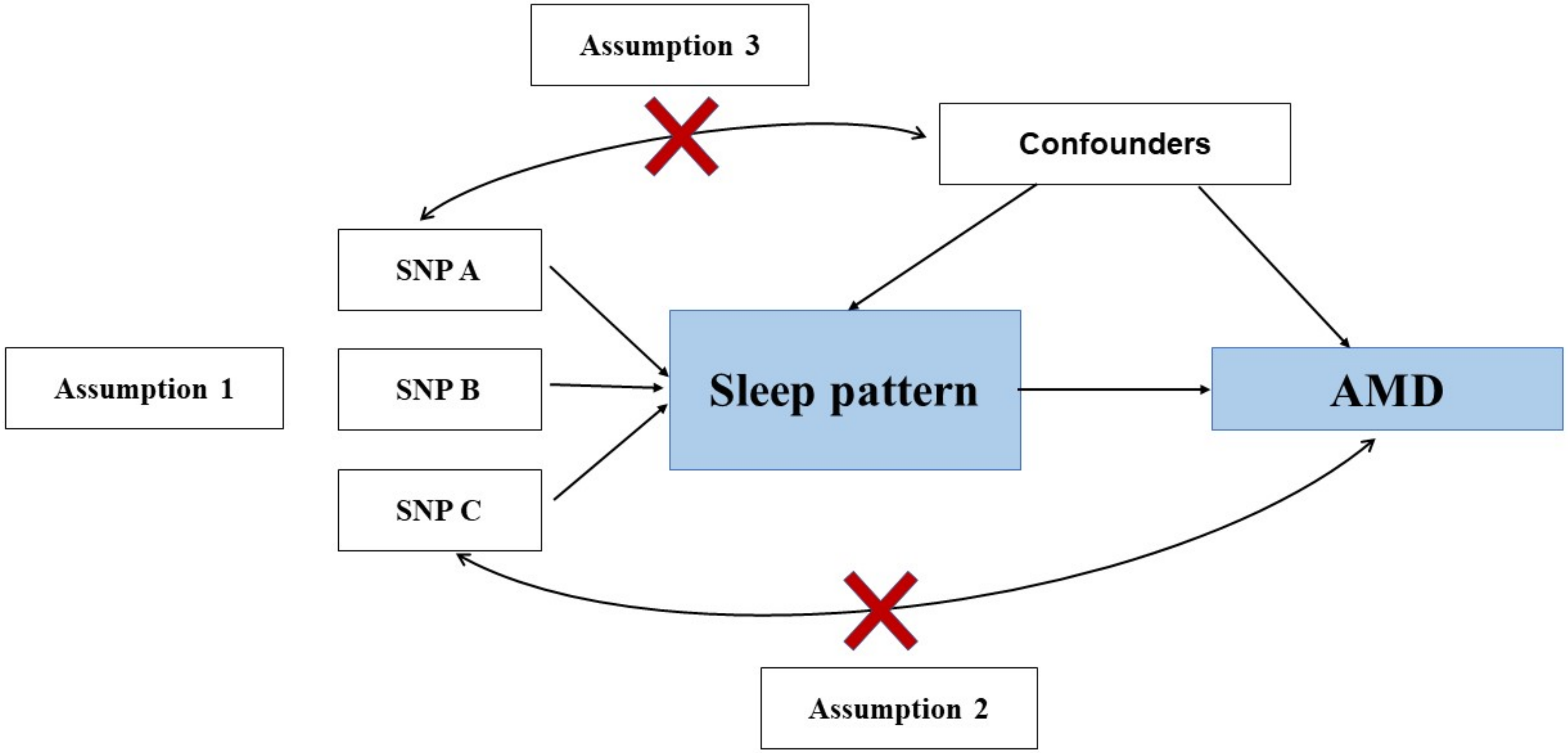

Supplementary Figure 1. Principles of MR and the assumptions required to obtain an unbiased causal effect estimate

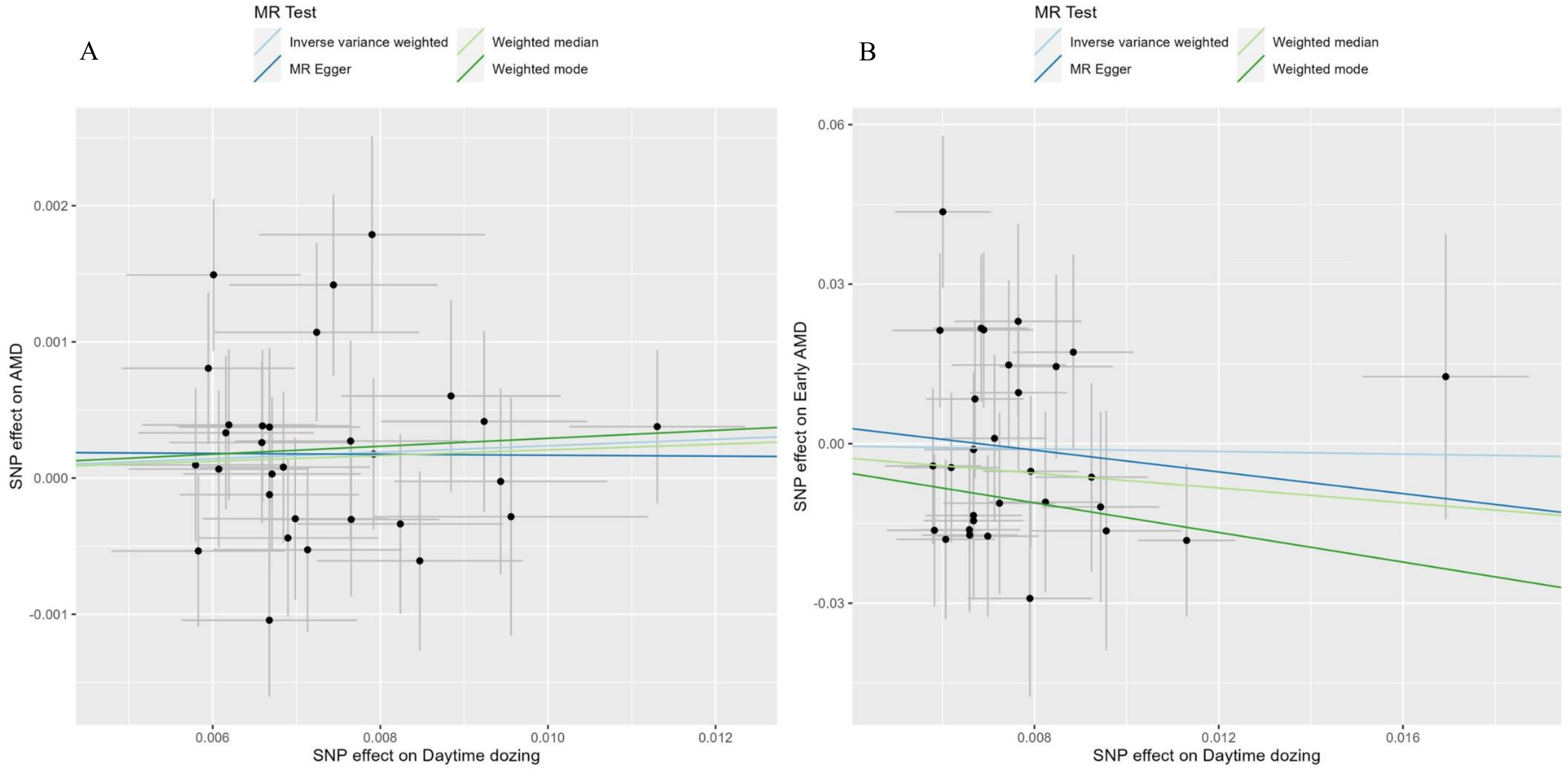

Supplementary Figure 2. Scatter and leave-one-out plots of daytime dozing with the risk of AMD and early AMD.
Scatter plot demonstrating the effect of daytime dozing associated genetic variants on AMD on the log-odds scale. The slopes of each line represent the causal association for each method. (B) Scatter plot demonstrating the effect of daytime dozing associated genetic variants on early AMD on the log-odds scale. The slopes of each line represent the causal association for each method. Scatter plots were utilized to display per-allele association with outcome risk in relation to per-allele association with one standard deviation of exposure. Vertical and horizontal gray lines were included to show the 95% CI for each SNP.

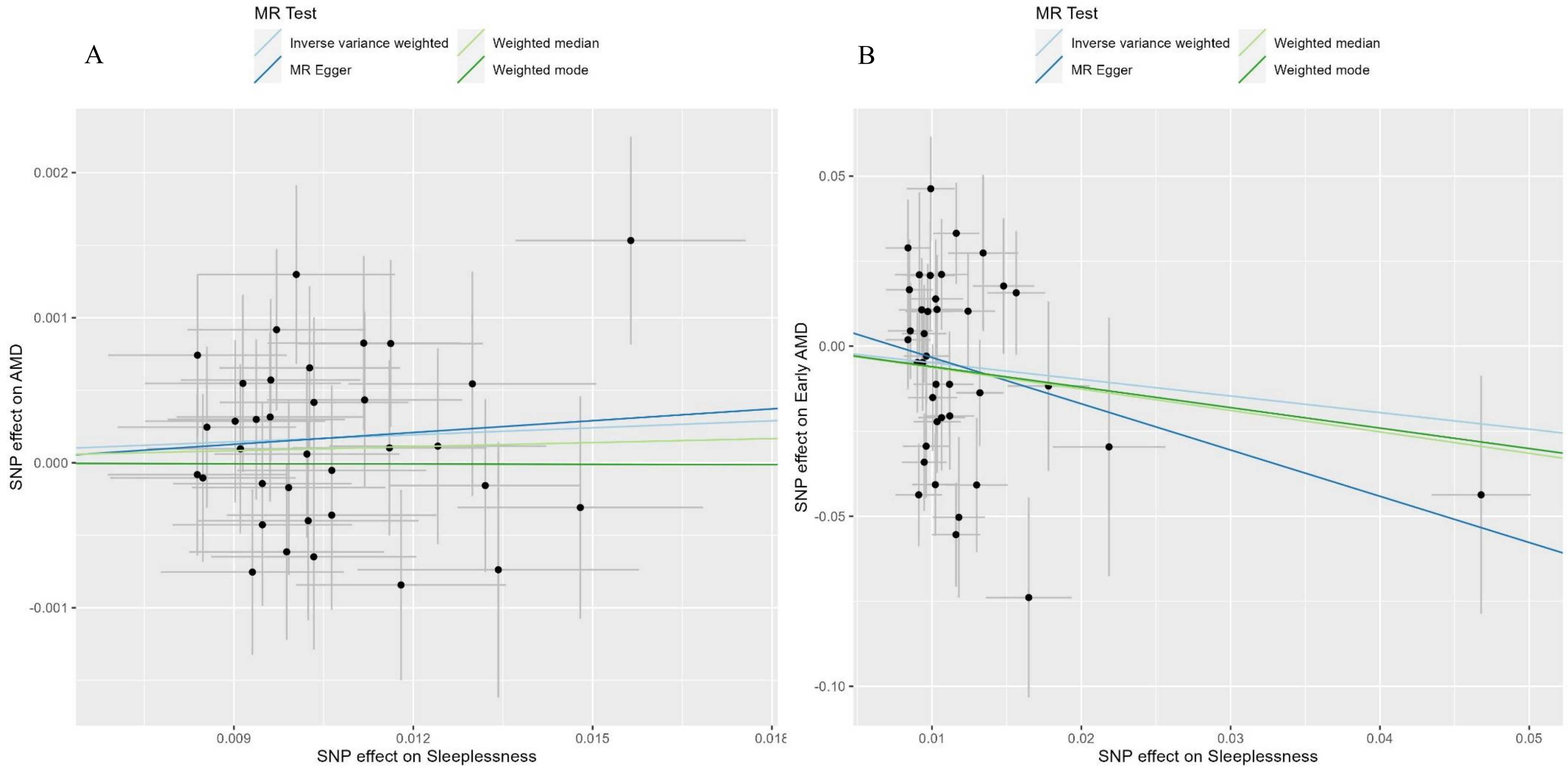

Supplementary Figure 3. Scatter and leave-one-out plots of sleeplessness with the risk of AMD and early AMD.
Scatter plot demonstrating the effect of sleeplessness associated genetic variants on AMD on the log-odds scale. The slopes of each line represent the causal association for each method. (B) Scatter plot demonstrating the effect of sleeplessness associated genetic variants on early AMD on the log-odds scale. The slopes of each line represent the causal association for each method. Scatter plots were utilized to display per-allele association with outcome risk in relation to per-allele association with one standard deviation of exposure. Vertical and horizontal gray lines were included to show the 95% CI for each SNP.
